# Safety and Efficacy of Flexible Ureteroscopy for Renal Stone Disease in Elderly Patients: A Scoping Review

**DOI:** 10.3390/jcm15041389

**Published:** 2026-02-10

**Authors:** Federico Falsetti, Valentina Maurizi, Luca Spinozzi, Enrico Sicignano, Savio Domenico Pandolfo, Michele Marchioni, Luigi Schips, Angelo Cafarelli

**Affiliations:** 1 Department of Urology, Casa di Cura Villa Igea, 60127 Ancona, Italy; federicofalsetti92@gmail.com (F.F.);; 2 Internal Medicine Unit Jesi, Area Vasta 2 ASUR Marche, 60035 Jesi, Italy; 3 Department of Medical and Surgical Sciences (DIMEC), University of Bologna, 40138 Bologna, Italy; 4 Department of Neurosciences and Reproductive Sciences and Odontostomatology, University of Naples “Federico II”, 80131 Naples, Italy; 5 Department of Urology, University of L’Aquila, 67100 L’Aquila, Italy; 6 Department of Urology, Medical School, UniCamillus-Saint Camillus International University of Health and Medical Sciences, 00131 Rome, Italy

**Keywords:** flexible ureteroscopy, retrograde intrarenal surgery, renal stone disease, elderly patients, surgical outcomes

## Abstract

**Background**: The global incidence of urolithiasis is increasing, with a growing proportion of cases occurring in elderly patients. Flexible ureteroscopy (fURS) is an established minimally invasive treatment for renal stones; however, concerns persist regarding its safety and effectiveness in older populations with higher comorbidity burdens. This scoping review aims to evaluate the current evidence on the safety and efficacy of fURS in elderly patients, with a specific focus on perioperative complications, postoperative recovery, and long-term outcomes. **Methods**: A scoping review was conducted in accordance with the PRISMA Extension for Scoping Reviews (PRISMA-ScR). A systematic literature search of PubMed, Embase, Scopus, and the Cochrane Central Register of Controlled Trials was performed from database inception to 26 November 2025. Observational and experimental studies evaluating outcomes of fURS in elderly patients were included. Data were synthesized descriptively. **Results**: Fourteen studies met the inclusion criteria, comprising predominantly retrospective cohort analyses and large registry-based studies. Definitions of elderly patients varied across studies (≥65 to ≥80 years). Elderly patients consistently exhibited higher comorbidity burdens and ASA scores than younger cohorts. Intraoperative complications were uncommon (<5%) and largely independent of age. Overall postoperative complication rates ranged from 12% to 18%, with the majority being low grade (Clavien–Dindo I–II). Major complications (Clavien–Dindo ≥III) were rare, generally occurring in fewer than 5% of cases. Stone-free rates were comparable between elderly and non-elderly patients. Long-term outcomes, including renal function preservation, stone-event-free survival, and late complications, were favorable and not significantly influenced by age. **Conclusions**: Current evidence indicates that fURS is a safe and effective treatment option for renal stone disease in elderly patients. Chronological age alone should not be considered a contraindication to ureteroscopic stone surgery. Careful patient selection, perioperative optimization, and procedural efficiency remain critical determinants of outcomes.

## 1. Introduction

Urolithiasis is a highly prevalent condition with a rising global incidence, a trend attributed in part to population aging and the increasing burden of metabolic comorbidities [1]. Although historically considered a disease typical in middle-aged adults, kidney stone disease has become increasingly common among individuals over 65 years of age. This shift is influenced by age-related metabolic alterations, dietary factors, pharmacological exposures, and a higher prevalence of systemic comorbidities [2,3]. In this age group, elderly patients are estimated to account for 9.6% to 16% of all stone formers, with a reported lifetime prevalence of approximately 14% [4].

Flexible ureteroscopy (fURS) has emerged as a cornerstone in the management of renal calculi, particularly for stones measuring less than 20 mm. Current European Association of Urology guidelines recommend ureteroscopy as a first-line or alternative treatment to shock-wave lithotripsy, depending on stone characteristics and patient-related factors, owing to its high efficacy and acceptable morbidity profile [5]. In recent years, fURS has demonstrated favorable stone-free rates ranging from 75% to 90%, even in technically challenging cases [6,7]. Moreover, large series and systematic reviews have reported overall complication rates of approximately 9% to 15%, while major complications (Clavien–Dindo grade ≥ III) remain uncommon, typically occurring in fewer than 3% of cases [8].

Growing evidence suggests that ureteroscopy can be performed safely and effectively in elderly patients when appropriate perioperative management is employed. Comparative studies across age groups indicate that older individuals achieve satisfactory stone-free and perioperative safety, although the morbidity index is a predictive factor of postoperative complications and survival [9]. Elderly patients represent a clinically distinct subgroup beyond chronological age alone. This population is frequently characterized by increased frailty, reduced physiological reserve, polypharmacy, and a higher burden of cardiovascular, metabolic, and renal comorbidities. These factors may influence perioperative management and postoperative recovery by increasing vulnerability to anesthetic stress, infectious complications, fluid imbalance, and renal function deterioration [10].

In the context of an expanding elderly population and the increasing utilization of ureteroscopy, it remains clinically relevant to clarify whether advanced age should influence treatment selection or represent a limiting factor. This scoping review, therefore, aims to synthesize the current evidence regarding the safety and efficacy of flexible ureteroscopy for renal stones in elderly patients, with a particular focus on perioperative outcomes, complication profiles, and longer-term results.

## 2. Evidence Acquisition

### 2.1. Literature Search

This scoping review adhered to the PRISMA Extension for Scoping Reviews guidelines [11]. A comprehensive search of Embase, PubMed, the Cochrane Central Register of Controlled Trials, and Scopus was conducted on 26 November 2025, covering all records from database inception. The following search terms and Boolean operators were applied: (elderly OR older OR geriatric OR “aged” OR “older adults” OR “advanced age”) AND (“flexible ureteroscopy” OR “flexible ureterorenoscopy” OR “retrograde intrarenal surgery” OR RIRS) AND (“renal stone*” OR “kidney stone*” OR nephrolithiasis OR urolithiasis OR “upper urinary tract stone*” OR “renal calculi”).

### 2.2. Selection Criteria

The clinical question was structured according to the PICOS (Population, Intervention, Comparison, Outcomes, Study design) framework:

- Population: Elderly patients with renal or upper urinary tract stones.
- Intervention: Retrograde intrarenal surgery (RIRS)
- Comparison: Younger/non-elderly patients or different elderly subgroups who underwent RIRS
- Outcomes: The primary outcomes are Length of stay, Intraoperative complications, Perioperative complications, and Long-term complications. Secondary outcomes included the Stone-free rate (SFR).
- Study Design: Observational and experimental studies.

### 2.3. Study Screening and Selection

Studies were included if they met the PICOS criteria. Exclusion criteria encompassed non-English publications, reviews, letters to the editor, conference abstracts, and case reports. Eligible designs included retrospective, prospective, and prospective randomized studies. Two independent reviewers screened all retrieved records using Covidence systematic review software (https://www.covidence.org/, Veritas Health Innovation, Melbourne, Australia). Any discrepancies were resolved through consultation. Full-text articles were assessed for eligibility based on relevance to the review’s objectives. This review was registered on the Open Science Framework: https://osf.io/upk4t accessed on 3 January 2026.

## 3. Results

### 3.1. Literature Screening

**Figure 1:**
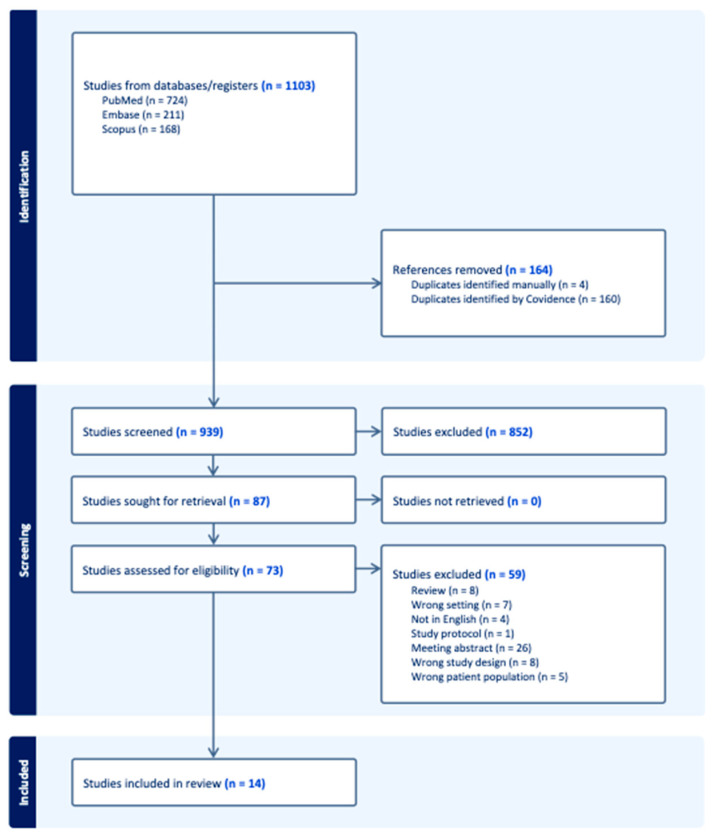
PRISMA 2009 flow diagram.

The preliminary literature search yielded 1103 articles. Following the removal of 164 duplicate entries, 939 studies remained for screening. Title and abstract screening resulted in the exclusion of an additional 852 articles that did not meet the relevance criteria for the present study. Full-text assessment was conducted on the remaining 87 studies, of which 73 were excluded based on predefined eligibility criteria. Ultimately, 14 studies were deemed eligible and incorporated into the final analysis. The study selection process is illustrated in the 2020 PRISMA flow diagram (Figure 1), Table S1 see the Supplementary Materials.

### 3.2. Study Characteristics

The dataset comprised thirteen retrospective studies [12,13,14,15,16,17,18,19,20,21,22,23,24], the main characteristics of the included studies are summarized in Table 1. Definitions of elderly patients varied substantially across the included studies. Several studies defined elderly status as ≥65 years [15,16,18], whereas others adopted thresholds of ≥70 years [12,17], ≥75 years, or ≥80 years, particularly in analyses focusing on “old-old” and “oldest-old” populations [19], although stratified outcome reporting by age category was not consistently available across all studies. Across studies, elderly patients consistently demonstrated a higher burden of systemic comorbidities. Higher ASA scores (III–IV) were reported in approximately 30–55% of elderly patients, compared with 10–25% of younger controls [18,22,23]. Despite these differences, stone characteristics were generally comparable between age groups, with mean stone size typically ranging from 10 to 18 mm, and most stones located in the renal pelvis, calyces, or proximal ureter. Across the included studies, surgical outcomes were generally reported in aggregate and were not stratified according to stone location. Although some cohorts included upper urinary tract stones, including proximal ureteral calculi, outcomes were consistently presented as global measures without separate analyses for renal and ureteral stone location [13,19,20,22]. Several studies reported comorbidity burden using ASA score or comorbidity indices; however, only a limited number of multicenter registry-based studies performed multivariable or propensity score-adjusted analyses [21,23,24].

### 3.3. Intraoperative Complications

Across all included studies, intraoperative complications during flexible ureteroscopy were infrequent, predominantly minor, and not significantly associated with advanced age. In a large propensity-score-matched multicenter analysis, intraoperative complications occurred in 3.2% of elderly patients compared with 2.8% of non-elderly patients, with no statistically significant difference. Reported events included minor ureteral mucosal injury, transient bleeding impairing visualization, and difficulty in ureteral access, none of which required conversion to open surgery or resulted in long-term sequelae [23].

Data from a large international registry demonstrated intraoperative adverse events in 4.6% of elderly patients. These events included access sheath-related ureteral trauma, minor ureteral perforation without extravasation, and technical difficulties leading to early termination of the procedure. Multivariable analysis confirmed that age was not an independent predictor of intraoperative complications, whereas stone complexity and operative duration were significant risk factors [22].

Single-center cohort studies yielded consistent findings. Intraoperative complication rates of approximately 5% were reported in elderly patients, compared with 4–5% in younger cohorts, with complications limited to minor ureteral wall injury and self-limited bleeding. No ureteral avulsions or major vascular injuries were reported [18]. Other comparative studies similarly reported no cases of ureteral perforation or avulsion, with overall intraoperative complication rates below 5% regardless of age [16].

In studies focusing on very elderly populations, intraoperative complications occurred in approximately 3.3% of patients aged ≥80 years, with events restricted to minor mucosal injury [20]. Additional single-center series reported no major intraoperative adverse events, including no anesthetic-related instability, in elderly patients undergoing holmium laser ureteroscopy [13].

Collectively, these findings indicate that intraoperative risks are low and largely independent of chronological age, supporting the technical safety of flexible ureteroscopy in elderly patients.

### 3.4. Perioperative Complications

Perioperative complications occurring within 30 days after surgery were reported more frequently than intraoperative events, but were predominantly low-grade and manageable with conservative treatment.

In the large propensity-score-matched study, overall postoperative complications occurred in 12.4% of elderly patients, compared with 11.7% of non-elderly patients [23]. The most common complications included postoperative fever (6.1%), urinary tract infection (4.3%), and transient hematuria (3.8%). Major complications (Clavien–Dindo ≥ III), such as urosepsis requiring intensive care or ureteral obstruction necessitating reintervention, occurred in less than 3% of elderly patients.

Registry-based data reported an overall complication rate of 15.3%, with the most frequent adverse events being febrile urinary tract infection (7.4%), acute urinary retention (4.1%), and persistent hematuria requiring prolonged catheterization (3.2%). Severe complications, including sepsis, acute kidney injury, or emergency drainage, were observed in only 2.5% of cases. Age was not independently associated with postoperative morbidity, whereas longer operative time and larger stone burden were significant predictors [22].

Several single-center studies corroborated these findings. Minor infectious complications occurred in approximately 9% of elderly patients, although no significant difference was observed in major complications compared with younger cohorts [18]. Other cohorts reported overall postoperative complication rates of approximately 14%, primarily consisting of urinary tract infection and hematuria, with no Clavien–Dindo grade IV or V events [15].

Additional studies reported postoperative fever or infection in 8%, transient hematuria in 6%, and urinary retention in 4% of elderly patients, with all cases managed conservatively [16]. Similar findings were reported in other single-center cohorts, in which postoperative complications were predominantly infectious or irritative, and major complications occurred in fewer than 5% of cases [14].

In very elderly populations, overall complication rates of approximately 18% were reported in patients aged ≥80 years, largely driven by minor infections and transient voiding difficulties. Severe complications remained rare (<4%), and no perioperative mortality was reported [20]. Other series involving elderly patients with high comorbidity burdens similarly documented major complication rates below 5% [21].

Hospital length of stay was consistently longer among elderly patients, with mean stays of 2–3 days compared with 1–2 days in younger cohorts. This difference was largely attributed to postoperative monitoring for infection, hematuria, or urinary retention, rather than severe complications. Readmission and reintervention rates within 30 days were uniformly low, generally below 5% across studies.

### 3.5. Long-Term Complications

Long-term complications following flexible ureteroscopy or RIRS were infrequently reported and uncommon across the included studies.

Long-term stone outcomes were specifically evaluated in comparative cohorts assessing stone-event-free survival, with no significant difference observed between elderly and younger patients. Five-year stone-event-free survival exceeded 80% in both age groups, and increasing age was not associated with a higher risk of stone recurrence or secondary intervention [19].

Functional outcomes were addressed in studies evaluating renal function after ureteroscopy. Stable renal function was reported in more than 90% of elderly patients during follow-up, with no clinically meaningful decline in estimated glomerular filtration rate [21]. Similar findings were reported in earlier cohorts, which demonstrated no significant long-term renal deterioration after ureteroscopic holmium laser lithotripsy [13].

Registry-based data further supported long-term safety, reporting delayed complication rates below 3%, including ureteral stricture formation, late-onset hematuria, and recurrent urinary tract infection. Age was not identified as an independent predictor of late adverse events [22]. More recent multicenter data evaluating same-session bilateral RIRS in patients aged ≥70 years demonstrated acceptable long-term outcomes without an increased risk of delayed morbidity [24].

### 3.6. Stone-Free Rates

SFRs following flexible ureteroscopy in elderly patients were consistently high and comparable to those observed in younger populations. Across the included studies, reported SFRs ranged from 70% to 90%, depending on stone burden, definition of stone-free status, and imaging modality used for assessment [12,13,14,15,16,17,18,19,20,21,22,23,24]. Most studies evaluated SFR using computed tomography or ultrasonography at 1–3 months postoperatively, with residual fragments ≤ 2–4 mm considered clinically insignificant. In comparative cohorts, SFRs did not differ significantly between elderly and non-elderly patients, with elderly cohorts achieving SFRs of approximately 75–85% after a single session [13,14,15,16,17,18,23]. Registry data reported SFRs between 76% and 83%, despite inclusion of patients with higher comorbidity burden and more complex stone presentations [21]. In studies focusing on very elderly patients (≥80 years), SFRs were slightly lower, ranging from 70% to 75%**,** but remained within an acceptable clinical range and were achieved without a corresponding increase in major complications [19]. Importantly, long-term analyses demonstrated that stone-event-free survival exceeded 80% at 5 years, with no significant difference between elderly and younger patients [20].

## 4. Discussion

### 4.1. Main Findings

This scoping review demonstrates that fURS is a safe and effective treatment modality for renal and upper urinary tract stones in elderly patients. Across retrospective cohorts, propensity score-matched analyses, and large international registries, elderly patients achieved stone-free rates comparable to younger populations, with low rates of major complications and acceptable perioperative morbidity [18,19,20,21,22,23]. These findings remained consistent even among very elderly patients and those with a high burden of comorbidities, supporting the concept that chronological age alone should not be considered a contraindication to ureteroscopic stone surgery.

One of the most consistent observations across the included studies is that procedure-related variables outweigh age as predictors of adverse outcomes. Operative time, stone burden, stone complexity, and intrarenal pressure dynamics were repeatedly identified as key drivers of intraoperative and postoperative complications, whereas age itself was not an independent risk factor [22,25]. This aligns with previous analyses demonstrating that prolonged operative duration and infectious risk factors—rather than chronological age—are the principal predictors of postoperative urosepsis following ureteroscopy [26,27,28]. These findings are particularly relevant in elderly patients, who frequently present with higher ASA scores and multiple comorbidities. Despite this, rates of major complications (Clavien–Dindo ≥ III) remained consistently below 3–5% across the studies included in this review [18,19,20,21,22]. This suggests that appropriate perioperative optimization and operative efficiency mitigate the physiological vulnerability associated with aging, allowing elderly patients to benefit from minimally invasive stone surgery.

Technological advances in RIRS have enhanced procedural safety and efficiency [29]. Among recent innovations, suction-assisted ureteroscopy represents one of the most impactful developments for improving safety. A comprehensive scoping review by Yuen et al. summarized experimental and clinical evidence demonstrating that suction ureteral access sheaths significantly reduce intrarenal pressure, improve visibility, and facilitate active fragment evacuation [30]. Reduced intrarenal pressure limits pyelovenous and pyelolymphatic backflow, mechanisms strongly implicated in postoperative infection and sepsis—complications of particular concern in elderly patients. Clinical data reinforce these mechanistic insights. A multicenter outcome analysis by Kwok et al. evaluating different sheath sizes for FANS confirmed efficient stone clearance without increased ureteral injury or major complications [31,32]. These findings indicate that suction-assisted systems can be safely applied across a broad spectrum of stone burdens and anatomical scenarios, including those encountered in elderly patients. Hu et al. reported favorable outcomes using a 7.5 Fr ultra-thin flexible ureteroscope combined with a tip-flexible suctioning ureteral access sheath, achieving high stone-free rates with an acceptable safety profile [33]. In parallel, a recent meta-analysis by Liu et al. demonstrated that flexible and navigable suction ureteral access sheaths were associated with superior efficacy and comparable safety when compared with traditional access sheaths during flexible ureteroscopy [34]. Collectively, these data suggest that suction-assisted RIRS may offer a meaningful safety advantage in elderly patients by simultaneously reducing intrarenal pressure, operative time, and infectious risk. Early clinical evaluations demonstrate favorable safety and efficacy, although elderly-specific data remain limited. Therefore, further studies are needed to achieve more robust data to draw definitive conclusions for these patients.

### 4.2. Future Perspectives

Further evidence supporting the future role of flexible ureteroscopy in elderly patients arises from the integration of multiple technological advancements. Robotic RIRS platforms have been developed to enhance procedural precision, surgeon ergonomics, and intrarenal stability, thereby improving scope control and reducing operator fatigue. These features may be particularly advantageous in elderly patients, who often present with complex renal anatomy or require prolonged procedures [35]. In parallel, advances in laser lithotripsy—most notably thulium fiber laser (TFL) technology—have demonstrated stone-free and complication rates comparable to those of conventional holmium:YAG systems, while achieving significantly shorter lasing and overall operative times [36]. Reduced operative duration is of particular relevance in older populations, as it may mitigate anesthesia-related risks and indirectly decrease the likelihood of postoperative infectious complications, which remain a major concern in frail and comorbid patients [36,37]. Collectively, these technological refinements suggest that fURS is likely to become an increasingly safe, efficient, and tailored treatment option for elderly patients, warranting further prospective studies focusing on geriatric-specific outcomes.

### 4.3. Limitations

This scoping review has several limitations that should be acknowledged. First, the included evidence is predominantly derived from retrospective observational studies, which are inherently subject to selection bias, unmeasured confounding, and heterogeneity in data reporting. Second, there was substantial variability in the definition of “elderly” across studies, ranging from 65 to 80 years and above, limiting direct comparability and precluding age-stratified quantitative synthesis. Furthermore, definitions of stone-free status varied across studies, depending on the imaging modality used, with computed tomography, ultrasonography, or plain radiography leading to potential heterogeneity and overestimation of stone-free rates. However, this limitation has limited impact on the present review, which primarily focuses on safety outcomes rather than on comparative efficacy. Third, outcome definitions—particularly stone-free status, complication reporting, and follow-up duration—were inconsistent among studies, reducing the ability to draw firm conclusions regarding comparative effectiveness. Finally, although technological advancements such as suction-assisted ureteroscopy and novel laser systems were discussed, elderly-specific comparative data for these innovations remain limited.

## 5. Conclusions

This scoping review suggests that fURS and RIRS are associated with favorable safety and efficacy profiles for the management of renal and upper urinary tract stones in elderly patients. Across the available observational evidence, elderly individuals achieved low rates of major complications and acceptable perioperative morbidity, with stone-free rates comparable to younger cohorts. Chronological age alone does not appear to be independently associated with worse perioperative or long-term outcomes, while procedural factors such as stone burden, operative time, and infection risk seem to play a more prominent role. Current observational evidence supports the use of fURS in selected elderly patients when appropriate perioperative optimization is ensured. However, given the exclusively observational nature of the included studies, causal inferences cannot be drawn, and future prospective studies specifically designed to evaluate frailty-adjusted outcomes in elderly populations are warranted.

## Figures and Tables

**Table 1:** Summary of Studies Evaluating Flexible Ureteroscopy/RIRS in Elderly Patients.

First Author (Year)	Country	Study Design	Mean Age (Elderly, Years)	Stone-Free Rate	Follow-Up	Reported Complications	Authors’ Conclusions
Tolga-Gulpinar (2015) [12]	Turkey	Retrospective comparative cohort	68–70	75–85%	3 months	Fever, hematuria, irritative LUTS; major complications rare	RIRS safe and effective across age groups
Yoshioka (2016) [13]	Japan	Retrospective single-center cohort	72	≈80%	6–12 months	Minor fever and hematuria only	Holmium laser URS safe in elderly
Berardinelli (2017) [14]	Italy	Retrospective single-center cohort	70	78–85%	3–6 months	Mainly infectious/irritative; <5% major	RIRS feasible and safe despite comorbidities
Cakici (2019) [15]	Turkey	Retrospective comparative cohort	67	≈82%	3 months	UTI, hematuria, urinary retention; no Clavien IV–V	Comparable safety and efficacy to non-elderly
Ozgur (2020) [16]	Turkey	Retrospective comparative cohort	69	75–80%	3 months	Fever/UTI, hematuria, urinary retention; conservative management	RIRS efficient and safe in elderly
Aykac (2020) [17]	Turkey	Retrospective age-stratified cohort	71	78–88%	3 months	Minor complications predominated	RIRS safe regardless of age
Koras (2021) [18]	Turkey	Retrospective comparative cohort	68	80–90%	3 months	Minor ureteral injury, infection; no major difference	URS safe across age groups
Taguchi (2022) [19]	Japan	Retrospective cohort (oldest-old)	≥80	70–75%	3 months	Minor infection/voiding issues; severe < 4%	URS safe even in ≥80 years
Tamiya (2023) [20]	Japan	Retrospective comparative cohort	71	>80% stone-event–free survival	60 months	No age-related increase in complications	Age does not impair long-term outcomes
Giulioni–FLEXOR (2023) [21]	Multinational	Multicenter registry	72	76–83%	3 months	Febrile UTI, retention; Clavien ≥ III 2.5%	Age not independent predictor of outcomes
Solomon (2023) [22]	Israel	Retrospective single-center cohort	74	≈85%	12 months	Major complications < 5%; renal function preserved	Good efficacy and functional outcomes
Akgül–RIRSearch (2024) [23]	Multinational	Propensity-matched multicenter study	70	80–85%	3 months	Fever, UTI; Clavien ≥ III <3%	RIRS safe after comorbidity adjustment
Giulioni (2025) [24]	Multinational	Multicenter bilateral RIRS cohort	≥70	70–80%	6–12 months	Acceptable morbidity; low delayed complications	Bilateral RIRS feasible in selected elderly

## Data Availability

No new data were created or analyzed in this study.

## Supplementary Materials

The following supporting information can be downloaded at: https://www.mdpi.com/article/10.3390/jcm15041389/s1, Table S1: Safety and Efficacy of Flexible Ureteroscopy for Renal Stone Disease in Elderly Patients: A Scoping Review (PRISMA-ScR) Checklist [11].
